# Cladribine repurposed in multiple sclerosis: making a fortune out of a generic drug

**DOI:** 10.1136/ejhpharm-2019-002026

**Published:** 2019-08-30

**Authors:** Hans J C Buiter, Luc Derijks, Chris J J Mulder

**Affiliations:** 1 Department of Clinical Pharmacology and Pharmacy, Amsterdam University Medical Centres, Amsterdam, The Netherlands; 2 Department of Clinical Pharmacy and Pharmacology, Maxima Medical Centre, Veldhoven, The Netherlands; 3 Department of Gastroenterology and Hepatology, Amsterdam University Medical Centres, Amsterdam, The Netherlands

**Keywords:** Purine nucleoside analogues, cladribine, multiple sclerosis, subcutaneous, cost, drug, re-discovery, off-label, immune suppression, DMT

Cladribine (CdA), a purine nucleoside analogue (PNA) that targets anti-CD4 and 8 T-cells, has recently been repositioned by Merck as an oral disease-modifying therapy for of highly active relaspe-remitting Multiple Sclerosis (RRMS), available as oral cladribine tablets (Mavenclad 10 mg). Its surplus value in the existing panel of disease-modifying therapy (DMT) for MS like the anti-CD20 B-cell targeting monoclonal antibodies, that is, rituximab (mouse chimeric), ocrelizumab (humanised) and ofatumumab (fully human) of which present data suggest that these are very effective in multiple sclerosis, is curious.^1^ In this personal viewpoint, we would like to highlight the potentially usefulness of PNA’s available and their limitations.

**Figure 1:**
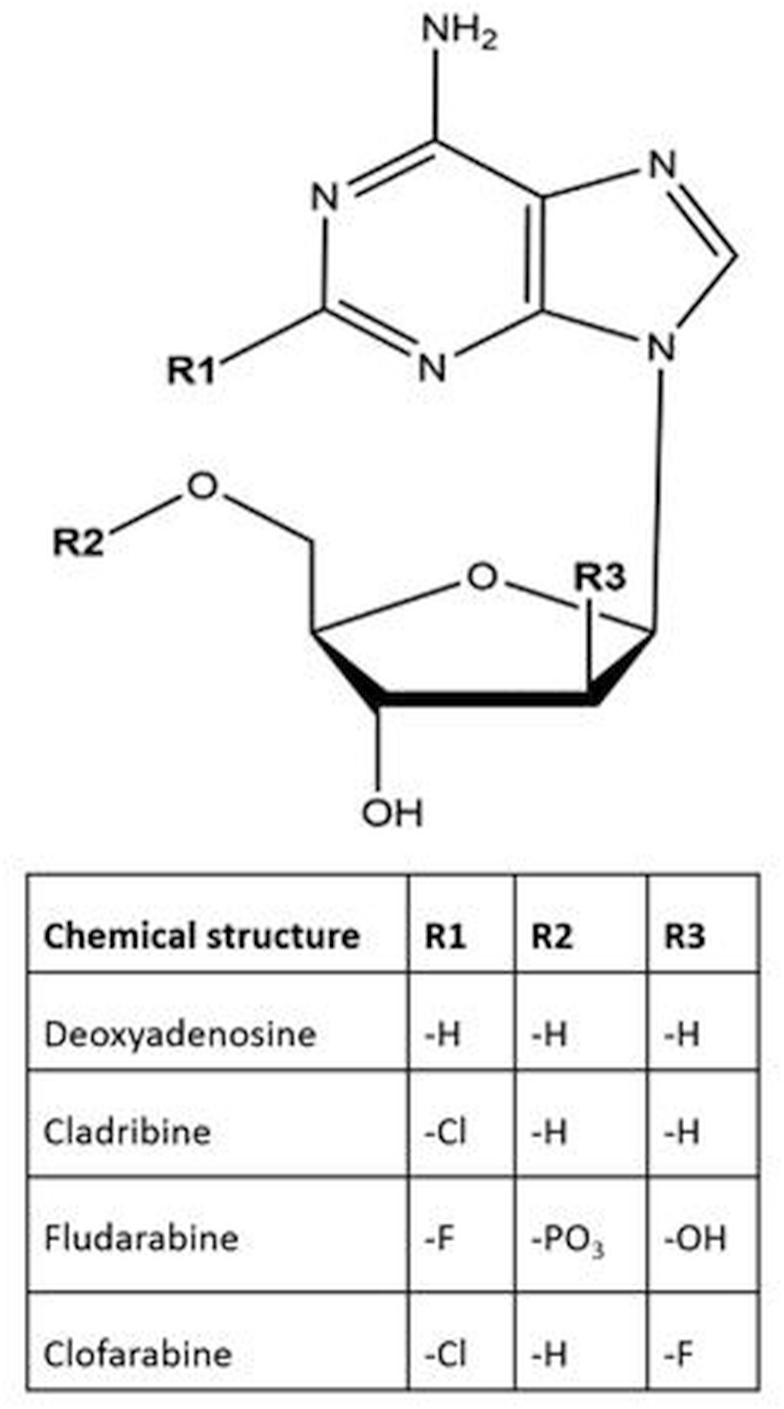
Chemical structures of purine analogues cladribine, fludarabine and clofarabine, compared with their natural deoxynucleoside, deoxyadenosine.

PNAs are active in chronic lymphocytic leukaemia, hairy cell leukaemia (HCL) and off-label in low-grade lymphomas. Cladribine has been used for HCL since the early 1980s as intravenous therapy.^2^ Cladribine delivered subcutaneously (SC) appeared to be most convenient in HCL and is considered to have equal efficacy compared with intravenous administration. Oral CdA use has been suggested since the early 1990s by Carson *et al*^3^ were it not that being unstable at acidic pH and is degraded by bacterial nucleoside phosphorylases. Other available PNAs are fludarabine (F-Ara) and clofarabine (CAFdA), which all are deoxyadenosine derivatives that act as antimetabolites that compete with natural deoxynucleosides used for DNA synthesis (figure 1).

All of these PNAs need to be metabolised to exert their cytotoxic effect to lymphoid cells. Because of this metabolism pathway and transportation by specialised nucleoside membrane transporters and subsequent phosphorylation to their active corresponding nucleotide, they are supposed to be especially active against low-grade malignancies with similar toxicity profiles for above-mentioned diseases that include moderate to profound and prolonged immunosuppression, thus being clinically effective in haematological malignant disorders and autoimmune disorders, including RRMS.^4^ It is worth to note that CAFdA was developed as a rational extension of the deoxyadenosine analogues to overcome the per oral bioavailability limitations and incorporate the best qualities of both F-Ara and CdA while having a similar metabolic/toxicity profile.

The prolonged immunosuppression by PNAs can indeed be beneficial for controlling relapsing remitting MS. As was shown for fludarabine, which was investigated as adjunct therapy in interferon-(beta)-treated RRMS.^5^ Preliminary interim analyses suggest that temporary fludarabine therapy may provide sustained immunosuppression. Cladribine performs similar as fludarabine and was first licenced for RRMS in 2011, yet later withdrawn when regulators requested more studies to address issues related to severe lymphopaenia. After the registration of alemtuzumab for relapsing remitting MS, which induces significantly more lymphopaenia and side effects than CdA, resubmission of CdA tablets was prompted to the regulators.^6^ Intriguingly, in the first observational pilot studies for MS, CdA was given intravenously.^7^ To date, CAFdA has not yet been investigated as a DMT for MS.

In general, oral drugs cost equal, usually less than parenteral drugs. As is the case for fludarabine, where intravenous versus oral drug costs per milligram are similar, that is, €2.57 versus €2.77 per mg. Apparently not for cladribine. Interestingly, the price of oral CdA with the registered indication for RRMS is over 20 000 euro per patient per year compared with less than 1000 euro per patient per year for equivalent dosing by parenteral administration.

In our opinion, drug industry strives to optimise profits by selecting markets where they can easily obtain a monopoly position while ensuring adequate drug production to meet market needs. We give two examples.^8^ In 2010, Valeant Pharmaceuticals acquired the rights to Syprine (trientine dihydrochloride), a drug from the 1960s used to treat Wilson’s disease. They raised its price substantially, by more than 3000% for a monthly supply: from $652 to $21 267. In 2015, Turing Pharmaceuticals acquired the rights to Daraprim (pyrimethamine), a drug approved by the Food and Drug Administration (FDA) in 1953 for toxoplasmosis. Turing, then the only manufacturer of pyrimethamine, raised the price of Daraprim by more than 5000% for one tablet: from $13.50 to $750. Oral CdA formulation is being marketed as Mavenclad in the Netherlands for list price of €2785 per 10 mg tablet compared with €283 for a 10 mg ampoule, taking into account that cladribine as active pharmaceutical ingredient costs only approximately €9 per mg. Therefore, it seems to us that Merck, by registering and marketing oral CdA (Maviclad) for RRMS is following a similar approach as described above and is mining Health Care to pay for this.

Obviously for patients, an oral formulation of a PNA seem to have clear benefits compared with parenteral formulations because of patient comfort and potentially less outpatient clinic admissions to receive PNA parenteral drug infusion. On the contrary, in general. compliance is better registered and controlled by parenteral drug administration at the outpatient clinic. In case of CdA, oral administration is not preferred per se because of its low bioavailability and interpatient variation; bioavailability of 10 mg oral CdA is approximately 40% (summary of product characteristics (SPC)). Parenteral CdA, for example, subcutaneous administration, would result in lower dosages due to higher bioavailability. Using subcutaneous cladribine instead of oral would potentially result in a total cost reduction 95% (Per Os: €32 500 vs SC €1600 based on the Mavenclad treatment scheme for RRMS for a patient with 70 kg bodyweight as described in the SPC).

In our opinion, the only PNA that would be beneficial for oral administration would be CAFdA, given its potential higher bioavailability compared with F-Ara and CdA.^9^ Unfortunately, an oral drug formulation of CAFdA is not yet available.

Drug repositioning or reprofiling/repurposing is the process of discovering, validating and marketing previously approved drugs for new indications. This process is of growing interest to academia and industry because of reduced time and costs associated with developing repositioned drugs. Newly licenced pharmaceutical indications are frequently approved without any controlled trial results, particularly in solid and haematological malignancies. Cladribine has been available off label and reported in neurological journals since the early 1990s and has been well studied both orally and parenterally. However, only orally administered CdA has been licenced for the treatment of RRMS. Subcutaneous CdA for MS was originally developed for compassionate use. As such, it has only been administered to MS patients in few centres around the world.

Therefore, we would like to suggest to hospital pharmacists to promote the use of equivalent subcutaneous dosing with PNA therapy to their healthcare providers, like haematologists do worldwide, especially for CdA when considered for MS patients, as this will result in a 95% cost reduction, better bioavailability and less interpatient variation for their patients.
